# Clearance of Gram-Negative Bacterial Pathogens from the Ocular Surface by Predatory Bacteria

**DOI:** 10.3390/antibiotics10070810

**Published:** 2021-07-03

**Authors:** Eric G. Romanowski, Shilpi Gupta, Androulla Pericleous, Daniel E. Kadouri, Robert M. Q. Shanks

**Affiliations:** 1 Charles T. Campbell Eye Microbiology Laboratory, Department of Ophthalmology, The Eye and Ear Institute, University of Pittsburgh School of Medicine, 203 Lothrop Street, Room 1020, Pittsburgh, PA 15213, USA; romanowskieg@upmc.edu; 2 Department of Oral Biology, Rutgers School of Dental Medicine, 110 Bergen St, Newark, NJ 07103, USA; sgupta1109@gmail.com (S.G.); androulla830@gmail.com (A.P.); kadourde@sdm.rutgers.edu (D.E.K.)

**Keywords:** ocular infection, predatory bacteria, *Bdellovibrio*, *Micavibrio*, *Pseudomonas aeruginosa*, *Serratia marcescens*, conjunctivitis, keratitis

## Abstract

It was previously demonstrated that predatory bacteria are able to efficiently eliminate Gram-negative pathogens including antibiotic-resistant and biofilm-associated bacteria. In this proof-of-concept study we evaluated whether two species of predatory bacteria, *Bdellovibrio bacteriovorus* and *Micavibrio aeruginosavorus*, were able to alter the survival of Gram-negative pathogens on the ocular surface. Clinical keratitis isolates of *Pseudomonas aeruginosa* (strain PAC) and *Serratia marcescens* (strain K904) were applied to the ocular surface of NZW rabbits followed by application of predatory bacteria. At time intervals, surviving pathogenic bacteria were enumerated. In addition, *B. bacteriovorus* and *S. marcescens* were applied to porcine organ culture corneas under contact lenses, and the ocular surface was examined by scanning electron microscopy. The ocular surface epithelial layer of porcine corneas exposed to *S. marcescens*, but not *B. bacteriovorus* was damaged. Using this model, neither pathogen could survive on the rabbit ocular surface for longer than 24 h. *M. aeruginosavorus* correlated with a more rapid clearance of *P. aeruginosa* but not *S. marcescens* from rabbit eyes. This study supports previous evidence that predatory bacteria are well tolerated by the cornea, but suggest that predatory bacteria do not considerably change the ability of the ocular surface to clear the tested Gram-negative bacterial pathogens from the ocular surface.

## 1. Introduction

Predatory bacteria including *Bdellovibrio bacteriovorus* and *Micavibrio aeruginosavorus* are Gram-negative bacteria that prey upon other Gram-negative bacteria [1,2]. These species are able to prey on a wide range of antibiotic-resistant bacteria including many human pathogens [3,4] such as ocular isolates of *Pseudomonas aeruginosa* and *Serratia marcescens* [4]. *B. bacteriovorus* has a broad host-range by which it invades the bacterial cell and replicates in the bacterial periplasm, whereas *M. aeruginosavorus* exhibits a narrower host-range and acts as an epibiotic predator as it attaches to the outside of prey bacteria [5]. These predators were shown to be highly effective against bacteria in biofilms, which are notoriously recalcitrant to traditional antibiotic therapy [6,7,8,9].

We previously postulated that predatory bacteria could be used as a topical treatment for bacterial infection of the eye and demonstrated that predatory bacteria are not toxic to human ocular surface cell lines and well tolerated on the ocular surface of rabbits [4,10]. Other groups have found similar tolerability of predatory bacteria on leporine and bovine ocular surfaces [11,12]. Furthermore, intravenous and intranasal inoculation of *Micavibrio* and *Bdellovibrio* species, even at high numbers, caused no morbidity or mortality in mice, although they did mildly increase production of proinflammatory cytokines IL-6, TNF-alpha, and chemokine CXCL-1 [13], and numerous mammalian cell lines were unperturbed by *Bdellovibrio* strains [4,14,15]. Together, these data suggest that *Bdellovibrio* and *Micavibrio* species can be safely used as an experimental therapeutic. Additionally, in vivo studies had reviled that predatory bacteria have potential as “living antimicrobials” for control of pathogens. *B. bacteriovorus* have shown efficacy in limiting *Klebsiella pneumoniae* and *Yersinia pestis* proliferation in airway and systemic rodent infection models [16,17]. Similarly, they were able to prey upon *Shigella flexneri* in the hindbrain of zebrafish, promoting the survival of the zebrafish larvae [18].

Ocular infections caused by Gram-negative bacteria, such as keratitis, are associated with contact lens use and can lead to a loss of ocular acuity [19,20,21]. Leading causes of these infections include *Pseudomonas aeruginosa* and *Serratia marcescens* [22,23,24]. Antibiotic resistance has been noted among keratitis isolates and is correlated with worse clinical outcomes [24,25,26,27,28,29]. Due to the need for new approaches to treat resistant microbial infections, we evaluated the ability of *B. bacteriovorus* and *M. aeruginosavorus* to promote the clearance of keratitis isolates of *S. marcescens* and fluoroquinolone-resistant *P. aeruginosa* and from the ocular surface using a rabbit ocular surface occupancy model.

## 2. Results

### 2.1. Scanning Electron Microscopy Visualization of B. bacteriovorus 109J with Porcine Corneas Ex Vivo

**Figure 1:**
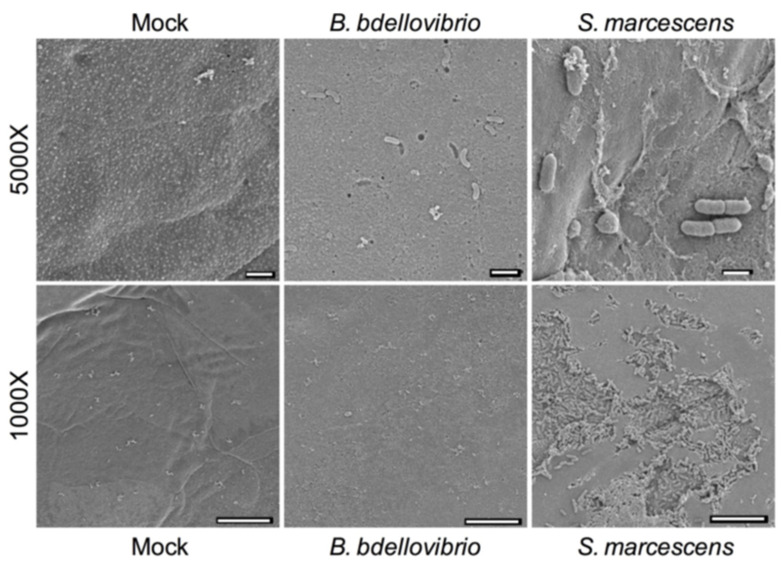
SEM micrographs of porcine corneal surfaces exposed to bacteria for 3 h ex vivo. Representative images are shown. Top row bars, 1 µm. Bottom row bars, 10 µm. Both *B. Bdellovibrio* strain 109J and *S. marcescens* strain K904 could adhere to the corneal surface, but *S. marcescens* was associated with damage to the epithelium.

As a first step in the study we visualized the interaction of predatory bacteria and the cornea in order to determine whether predatory bacteria could adhere to the corneal surface and whether there was any clear impact of this interaction using *B. bacteriovorus* 109J as a representative strain of predatory bacteria. Although a previous study demonstrated the absence of a clinical inflammatory response by rabbits, it did not evaluate the ocular surface at a microscopic level. Figure 1 depicts ex vivo porcine corneas from an organ culture model where *B. bacteriovorus* strain 109J was in contact with the ocular surface under a contact lens for 3 h. The predatory bacteria could adhere to the ocular surface, but failed to produce any clear epithelial damage similar to the mock treated (no bacteria) samples. By comparison, when using a sample Gram-negative pathogen, *S. marcescens* strain K904, under the same experimental conditions, adherent bacteria were present and were associated with erosion-like areas.

### 2.2. Clearance of Fluoroquinolone Resistant P. aeruginosa But Not S. marcescens from Rabbit Ocular Surfaces Was Facilitated by Instillation of Predatory Bacteria

**Figure 2:**
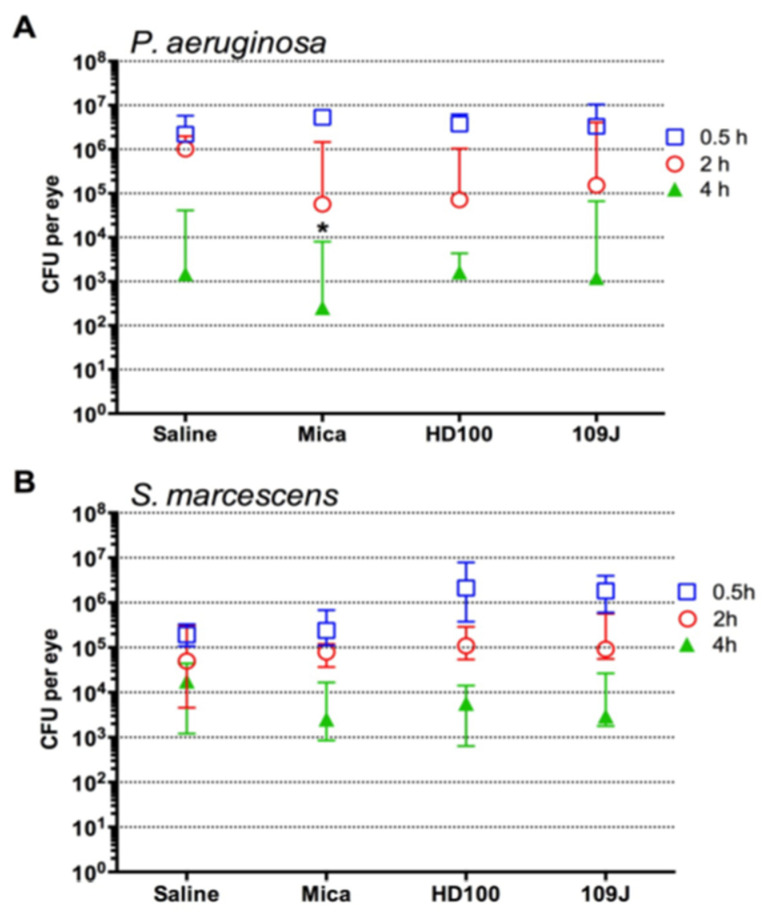
Predatory bacteria impact the ocular surface survival of *P. aeruginosa* and *S. marcescens*. (**A**,**B**). Medians and interquartile ranges of bacterial CFU from ocular surface of New Zealand white rabbits, (**A**). *P. aeruginosa*, *n* = 12 eyes per group. (**B**). *S. marcescens*, *n* = 8 eyes per group. Asterisks indicate significant differences from saline at the same time point (*p* < 0.05) as determined by Mann–Whitney test.

The survival of a fluoroquinolone-resistant keratitis isolate of *P. aeruginosa* (strain PAC) was evaluated on the ocular surface of NZW rabbits. PAC was previously shown to be susceptible to the predatory bacteria used in this study in vitro [4]. It was shown that PAC was reduced 2.13 Log_10_ CFU by *B. bacteriovorus* 109J, 3.91 Log_10_ CFU by *B. bacteriovorus* HD100 and 2.98 Log_10_ CFU by *M. aeruginosavorus* ARL-13. Predatory bacteria or saline was applied topically at 1, 3, and 5 h post-instillation of *P. aeruginosa*, and bacteria were enumerated at 0.5, 2, and 4, and 24 h (Figure 2A). No growth was measured from the samples taken at 24 h.

In the saline group, median PAC levels remained steady at over 10^6^ CFU for the first 2 h then dropped down to just under 1500 CFU per swab at 4 h. Notably between hour 0 and 2 there was no clear reduction in PAC in the saline treated eyes, whereas the predatory bacteria treated eyes had a reduction in the number of PAC bacteria. PAC CFU dropped 2.0, 1.7, and 1.3 Log_10_ for *B. bacteriovorus*109J, *Micavibrio* (Mica), and *B. bacteriovorus* HD100, respectively, between 2 and 4 h. PAC CFU from the Mica treatment group at 4 h post-inoculation (254 CFU) was significantly different than the saline treatment group (1483 CFU), (Mann–Whitney *p* < 0.05).

The same approach was performed with *S. marcescens* contact lens associated keratitis isolate K904, which is also susceptible to predatory bacteria in vitro [30]. Garcia et al. showed that *B. bacteriovorus* 109J reduced *S. marcescens* K904 CFU by 4.1 Log_10_ CFU but only 0.3 Log_10_ CFU by *M. aeruginosavorus* ARL-13 [30]. Here, we evaluated *S. marcescens* strain K904 predation by *B. bacteriovorus* HD100 in vitro and measured a 3.92 ± 0.17 Log_10_ CFU reduction compared to an increase of 0.17 ± 0.12 CFU change in the control samples without predatory bacteria (*n* = 3). *S. marcescens* CFU were similar at 0 and 2 h but were reduced at 4 h on the rabbit ocular surface (Figure 2B) compared with the saline control. Predatory bacteria did not significantly impact the survival of *S. marcescens* on the ocular surface.

## 3. Discussion

This study indicated that the predatory bacterium *B. bacteriovorus* strain 109J was not damaging to live corneas when tested in an organ culture model, which is consistent with previous studies indicating that predatory bacteria are well tolerated by tissue culture cell lines and mammals [10,12,14,15,16,18]. By contrast a representative ocular surface pathogen, *S. marcescens* caused clear damage to the corneal epithelia. This may be due to the many cytotoxic enzymes, such as PrtS, SlpB, and SlpE metalloproteases, and the pore-forming toxin ShlA, previously shown to be cytotoxic to ocular surface cells [31,32,33].

Two previous studies have evaluated the ability of predatory bacteria to reduce bacterial counts on the ocular surface; one with *Shigella flexneri* was inconclusive with respect to predation as the *S. flexneri* numbers were reduced following application of either *B. bacteriovorus* or non-pathogenic *Escherichia coli* [11]. In another study, lyophilized *B. bacteriovorus* strain 109J was used in topical treatment of calf corneas that had been infected with pathogenic *Moraxella bovis* using an infectious bovine keratoconjunctivitis model [12]. Boileau and colleagues concluded that the treatment group did not differ from the control group in treatment of the ocular surface infection [12]. By contrast, *B. bacteriovorus* treatment has been effective in reducing pathogen numbers in rat lung and zebrafish larvae hindbrain infection models [13,18] and reducing *Salmonella* numbers in the gut of chickens following oral dosing [34]. Therefore, it is clear that there are physiological limits to where predatory bacteria can be used as alternatives to antibiotics. The ocular surface is a notably hostile environment to bacteria and is considered paucibacterial with relatively few bacteria compared to other exposed mucosal surfaces [35]. Although DNA for Gram-negative bacteria have been isolated from the ocular surface following PCR amplification in several studies, it is not clear that they are constituents of the normal ocular microbiome, which is dominated by Gram-positive genera such as *Corynebacterium* and *Staphylococcus* that are resistant to the tested predatory bacteria [35,36,37]. Therefore, as was demonstrated for the rat gut microbiome [38], it is not expected that predatory bacteria would have a major effect on the ocular surface microbiome. Furthermore, *B. bacteriovorus* abundance has been positively correlated with a healthy gut microbiome and the absence of inflammatory disease in humans, suggesting a beneficial role for these organisms [39].

The act of swabbing or proparacaine topical anesthetic solution may have influenced the outcome of the study. Proparacaine has been shown to inhibit *Staphylococcus aureus*, but not *P. aeruginosa* growth in vitro [40]. However, a veterinary study demonstrated no significant effect of proparacaine on the number of bacteria isolated from ocular surface samples, suggesting that proparacaine did not impact this study [41]. Similarly, previous studies have demonstrated rapid clearance of *P. aeruginosa* on the ocular surface of rodents [42], suggesting that the rapid reduction of pathogen bacteria on the ocular surface was due to the innate immune system of the eye rather than due to swabbing the ocular surface.

There was a correlation of the presence of predatory bacteria with a reduction in *P. aeruginosa* CFU on the ocular surface at 2 and 4 h that only reached significance with *M. aeruginosavorus*. By contrast *S. marcescens* surface occupancy was not altered by the presence of predatory bacteria. The in vitro reduction of *S. marcescens* K904 (~4-log reduction) by *B. bacteriovorus* was higher than for *P. aeruginosa* PAC (2.1–3.9-log reduction); whereas *M. aeruginosavorus* reduced *P. aeruginosa* PAC (~3-log reduction) greater than *S. marcescens* K904 (~0.3-log reduction) in vitro [4,30]. While it is clear that these pathogens were preyed upon in vitro, whether there was active predation on the ocular surface was not formally determined in this pilot study. Indeed, predatory bacteria may stimulate the immune system to promote clearance of *P. aeruginosa*. Consistent with this hypothesis, in a zebrafish infection study, predatory bacteria preyed upon *S. flexneri* in the hind brain, but full clearance of the pathogen required both the predatory bacteria and the immune system [18]. On the ocular surface, colonization of *Corynebacterium* species can promote resistance to *Pseudomonas* infections that is dependent upon an IL-17 signaling mechanism [43]. It is possible that the predatory bacteria are invoking a similar protective immune response in rabbits.

Together, these data suggest that predatory bacteria are not damaging to the corneal epithelium and can influence the occupancy of pathogens on the ocular surface, but that they are not an effective method of clearing pathogens beyond that of the natural host defense systems.

## 4. Materials and Methods

### 4.1. Bacterial Strains and Culture

Keratitis isolates of *P. aeruginosa* strain PAC [44] and *S. marcescens* strain K904 [45] were used in this study. The *P. aeruginosa* strain was determined to be resistant to fluoroquinolone antibiotics (ciprofloxacin, gatifloxacin, ofloxacin, levofloxacin, and moxifloxacin) in a College of American Pathologists (CAP) and Clinical Laboratory Improvement Amendments (CLIA) certified microbiology laboratory following Clinical and Laboratory Standards Institute (CLSI) guidelines [44,46]. Susceptibility was interpreted using the CLSI (Clinical & Laboratory Standards Institute) serum standards and procedures for disk diffusion [46], and later determined using E-tests [47].

These bacteria were maintained in glycerol frozen stocks and were streaked to single colonies on TSA medium with 5% red blood cells (Blood agar) (Remel, Lenexa, KS) before use as described below. Bacteria were also cultured with lysogeny broth (LB) and LB with agar [48]. The predatory bacteria used in the study were *B. bacteriovorus* 109J [49] *B. bacteriovorus* HD100 (ATCC 15356) [50], and *M. aeruginosavorous* strain ARL-13 [51]. Predator lysates (cocultures) were prepared as reported previously [14,16]. In brief, *B. bacteriovorus* and *M. aeruginosavorus* were incubated with *E. coli* strain WM3064 (1 × 10^9^ CFU/mL) at 30°C for 24 and 72 h, respectively. The cleared lysates were filtered several times through a 0.45-μm Millex^®^-HV pore-size filter (Millipore, Billerica, MA, USA) in order to remove residual prey. Predators were washed and concentrated by sequential centrifugation cycles. The final predator pellets were re-suspended in Phosphate Buffered Saline (PBS) to reach final concentrations of 1 × 10^10^ PFU/mL *B. bacteriovorus* and 1 × 10^9^ PFU/mL *M. aeruginosavorus*.

### 4.2. Scanning Electron Microscopy

*B. bacteriovorus* strain 109J, prepared in PBS as described above, were applied in 50 µL samples to the surfaces of ex vivo porcine corneas and contact lenses (CL) were applied. PBS alone was used as a negative control, and *S. marcescens* strain K904 in PBS (3 × 10^9^) was applied as a control pathogen. Porcine eyes were obtained from Sierra Medical (Whittier, CA, USA) and corneal organ culture was performed as previously described but without antibiotics [52,53]. The ex vivo corneas were incubated at 37 °C for 3 h, then the CL were removed. The corneas were rinsed twice with PBS to remove non- or loosely adherent bacteria and fixed with glutaraldehyde (3%) overnight at room temperature. Corneas were then washed with PBS and post-fixed using aqueous osmium tetroxide (1%), dehydrated using increasing ethanol concentrations (30–100%), immersed in hexamethyldisilazane, air dried, and sputter coated with gold/palladium (6 nm). A JEOL JSM-6335F scanning electron microscope at 3 kV with the secondary electron imaging detector was used for imaging.

### 4.3. In Vitro Predation Assay

Susceptibility of *S. marcescens* strain K904 to *B. bacteriovorus* strain HD100 was tested as previously described [30]. HD100 and K904 were combined in 14 mL Falcon™ round-bottom polypropylene tubes by adding 0.4 mL of harvested predators (5 × 10^8^ PFU/mL) to 0.4 washed *S. marcescens* (4 × 10^9^ CFU/mL) and 1.2 mL HEPES buffer (HEPES at 25 mM supplemented with CaCl_2_ at 2 mM and MgCl_2_ at 3 mM). These were incubated at 30 °C on a rotary shaker set at 30 rpm. A control without *B. bacteriovorus* was included as a control. Colony forming units of *S. marcescens* were determined by dilution plating on LB agar plates after 24 of coculture. The experiment was repeated three times.

### 4.4. Rabbit Ocular Surface Occupancy Model

This study conformed to the ARVO Statement on the Use of Animals in Ophthalmic and Vision Research and was approved by the University of Pittsburgh Institutional Animal Care and Use Committee (Protocol 15025331). Female New Zealand white rabbits weighing 1.1–1.4 kg, were obtained from Charles River Oakwood rabbitry.

For the inocula, *P. aeruginosa* strain PAC and *S. marcescens* strain K904 were swabbed onto 5 blood agar plates and incubated at 37 °C overnight. Bacteria were scraped off the plates using a cotton tipped applicator and suspended in 5 mL of phosphate buffered saline (PBS) and adjusted to a culture density of OD_600_ of 5 in PBS. *P. aeruginosa* and *S. marcescens* inocula colony counts were determined using the EddyJet 2 spiral plating system (Neutec Group Inc., Farmingdale, NY, USA) on blood agar plates. The plates were incubated overnight at 37 °C for *P. aeruginosa* and 30 °C for *S. marcescens* and resulting colonies were enumerated using the automated Flash and Grow colony counting system (Neutec Group), with (~5 × 10^8^ CFU) in 50 µL samples of bacteria that were applied to the ocular surface of both eyes of unanesthetized rabbits. Fifty µL of the predatory bacteria were installed into the rabbits’ eyes and consisted of 2 × 10^8^ PFU/mL for *B. bacteriovorus* and 2 × 10^7^ PFU/mL for *M. aeruginosavorus*.

The ocular surfaces of both rabbit eyes were inoculated with *P. aeruginosa* (*n* = 12 rabbits) and *S. marcescens* (*n* = 8 rabbits). At 0.5, 2, 4, and 24 post-inoculation, each eye was cultured following topical anesthesia with 2 drops of 0.5% proparacaine (Proparacaine Hydrochloride Ophthalmic Solution, USP, 0.5%, Sandoz Inc., Princeton, NJ, USA) by inserting a Dacron-tipped applicator into the upper and lower fornices and gently manipulating the swab over the conjunctival and corneal surfaces. Swabs were placed into 1 mL of PBS and kept on ice. Dilutions (1:100 and 1:10,000) of the samples were made in PBS. The undiluted and diluted samples were plated on blood agar plates to enumerate bacteria as describe above. At 1, 3, and 5 h post-inoculation, 50 µL topical drops with predatory bacteria or saline were applied to eyes. *P. aeruginosa* and *S. marcescens* remaining of the ocular surface were enumerated as described above. Median colony forming units (CFU) were compared using non-parametric analysis with GraphPad Prism software.

### 4.5. Statistical Analysis

Mann–Whitney analysis was performed using GraphPad Prism statistical software version 6.0. *p*-values less than 0.05 were considered significant.

## Data Availability

Data are available on request.
